# Vessel-Associated Immune Cells in Cerebrovascular Diseases: From Perivascular Macrophages to Vessel-Associated Microglia

**DOI:** 10.3389/fnins.2019.01291

**Published:** 2019-12-04

**Authors:** Takashi Koizumi, Danielle Kerkhofs, Toshiki Mizuno, Harry W. M. Steinbusch, Sébastien Foulquier

**Affiliations:** ^1^Department of Neurology, Graduate School of Medical Science, Kyoto Prefectural University of Medicine, Kyoto, Japan; ^2^Department of Pharmacology and Toxicology, School for Mental Health and Neuroscience, Maastricht University Medical Center+, Maastricht, Netherlands; ^3^Department of Neurology, School for Cardiovascular Diseases, Maastricht University Medical Center+, Maastricht, Netherlands; ^4^Department of Pathology, School for Cardiovascular Diseases, Maastricht University Medical Center+, Maastricht, Netherlands; ^5^Department of Translational Neuroscience, School for Mental Health and Neuroscience, Faculty of Health, Medicine and Life Sciences, Maastricht University Medical Center+, Maastricht, Netherlands

**Keywords:** cerebrovascular dysfunction, neuroinflammation, cerebral small vessel disease, vascular cognitive impairment and dementia, microglia, macrophages, hypertension, stroke

## Abstract

Cerebral small vessels feed and protect the brain parenchyma thanks to the unique features of the blood–brain barrier. Cerebrovascular dysfunction is therefore seen as a detrimental factor for the initiation of several central nervous system (CNS) disorders, such as stroke, cerebral small vessel disease (cSVD), and Alzheimer’s disease. The main working hypothesis linking cerebrovascular dysfunction to brain disorders includes the contribution of neuroinflammation. While our knowledge on microglia cells – the brain-resident immune cells – has been increasing in the last decades, the specific populations of microglia and macrophages surrounding brain vessels, vessel-associated microglia (VAM), and perivascular macrophages (PVMs), respectively, have been overlooked. This review aims to summarize the knowledge gathered on VAM and PVMs, to discuss existing knowledge gaps of importance for later studies and to summarize evidences for their contribution to cerebrovascular dysfunction.

## Introduction

A growing body of evidence supports the importance of our immune system in disease progression, making the research community more aware of the complexity of disease’s mechanisms but offering at the same time new diagnostic and therapeutic opportunities. This holds true for cerebrovascular diseases such as cerebral small vessel disease (cSVD), the most prevalent cause of vascular cognitive impairment (Dichgans and Leys, 2017), in which the surroundings of brain small vessels are being scrutinized to decipher its exact pathophysiological mechanism. In this regard, perivascular immune cells have gained interest in the last three decades and both microglia and macrophages have been discussed in recent studies. The terms “perivascular microglia” and “perivascular macrophage,” given at several occasions, have not been always rightly used to describe immune cells associated with the cerebral vessels. Current state-of-the-art immunohistochemistry combined with confocal microscopy has revealed differential expressions of microglia/macrophage markers as well as morphological features that allow today a better discrimination of those brain perivascular immune cells.

In this review, the terms “vessel-associated microglia” (VAM) and “perivascular macrophages” (PVMs) will be first defined before summarizing the findings on VAM and PVMs associated with cerebrovascular diseases. This review aims to discuss the importance of differentiating VAM from PVMs. This emerging concept should be considered to fill in current research gaps in the field of neurodegenerative diseases involving cerebrovascular dysfunction.

## Historical Perspective

The first occurrence of the term “perivascular microglia” was in 1988, when Hickey and Kimura described the presence of bone marrow-derived cells located around cerebral vessels and expressing the cell surface glycoprotein ED-2 (Hickey and Kimura, 1988). However, Graeber et al. (1989) suggested that these ED-2-positive perivascular cells differ from microglia, which did not stain with ED-2 (Graeber et al., 1989) and he suggested to keep the term “perivascular microglia” for microglia located on the vicinity of vessels outside of the basal lamina (Graeber and Streit, 1990). The ED-2 antigen was later identified as CD163, a highly specific marker for PVMs, ruling out the possibility that these perivascular cells were pericytes (Fabriek et al., 2005, 2007; Table 1). From these early studies, a clear description of PVMs was made using their ED-2-positive immunoreactivity and their location within the perivascular space mainly around the large penetrating arterioles. After its introduction in 1990, however, the term “perivascular microglia” was used at many occasions instead of PVM and the term “juxtavascular microglia” was also found as an alternative for perivascular microglia (Lewis et al., 2009), overall creating a lot of confusion in this research field. To describe parenchymal microglia juxtaposed to the cerebral vasculature and outside of the glia limitans, we propose to avoid the use of the term “perivascular” and to refer to “VAM” to avoid any confusion with PVMs located in the perivascular space.

**TABLE 1:** Differentiation markers for microglia (MG), including vessel-associated microglia (VAM), and CNS macrophages (MP), including perivascular macrophages (PVMs).

**Marker**		**Functions**		**Gene expression**			**Immunohistochemistry**			**References**
**Gene**	**Protein**			**Immature**	**Adult**	**Injury/Inflammatory**	**Immature**	**Adult**	**Injury/Inflammatory**	
***Cd45***	CD45	T cell and B cell receptor- mediated activation	MG	Low	Low	Low				Goldmann et al., 2016; Martin et al., 2017; Rangaraju et al., 2018
			MP	High	High	High				
***Itgam***	CD11b (OX-42)	Cell adhesion; apoptosis; chemotaxis	MG	High	High	High				Robinson et al., 1986; Martin et al., 2017
			MP	High	High	High				
***Iba1/Aif1***	Iba-1/AIF-1	Complete functional profiles are unknown	MG/VAM	High	High	High	P (E9-)	P: high	P	Ito et al., 1998; Faraco et al., 2016
			MP	Low	Low	Low	P	P: weak	P	
***Cx3CR1***	Cx3CR1	Fractalkine receptor	MG	High	High	High	P (E8.5-)	P	P	Jung et al., 2000; Hammond et al., 2019
			MP	Low	Low	Low	P	P	P	
***Csf1r***	CSF1-R	Csf1(MCSF) receptor	MG	High	High	High	P	P	P	Akiyama et al., 1994; Raivich et al., 1998
			MP	Low	Low	Low	P	P	P	
***Tmem119***	TMEM119	A cell-surface protein of unknown function	MG	ND	High	Decrease	N: (-P3)	P	minor decrease	Bennett et al., 2016; Butovsky and Weiner, 2018; Furube et al., 2018; Jordão et al., 2019
			MP	ND	ND	ND	N	N	N	
***Sall1***	Sall1	A zinc-finger transcription factor	MG	High^*1^	High	High	P (E10.2-)^*1^	P	P	Buttgereit et al., 2016
			MP	ND	ND	ND	N	N^*2^	N	
***P2ry12***	P2RY12	Nucleotide receptor	MG	High	High	Low/ND	P (new born-)	P	major decrease	Mildner et al., 2017; Jordão et al., 2019
			MP	ND	ND	ND	N	N	N	
***Siglech***	Siglec-H	Sialic acid-binding cell surface lectin	MG	High	High	Stable∼decrease	P (E17)	P	P	Konishi et al., 2017; Baufeld et al., 2018; Butovsky and Weiner, 2018; Mrdjen et al., 2018
			MP	ND	ND	ND	N	N^*3^	N	
***Olfml3***	Olfml3	Proangiogenic factor	MG	High	High	Stable∼decrease	?	P	P	Baufeld et al., 2018; Neidert et al., 2018; Jordão et al., 2019
			MP	ND	ND	ND	?	N^*4^	N	
***Cd163***	CD163	Endocytosis; scavenger receptor	MG		ND	ND	?	N	P:occasionally	Fabriek et al., 2005; Pey et al., 2014
			MP		High	High	?	P^*5^	P^*6^	
***Mrc1***	CD206	Endocytosis; mannose receptor	MG		ND	low	?	N	P: occasionally	Holder et al., 2014; Goldmann et al., 2016; Bottcher et al., 2019
			MP		High	High	?	P	P	

*^∗^1: astrocytes and neuronal progenitor cells in the CNS during embryogenesis have high expression of sall1. ^∗^2: choroid plexus macrophages are negative, 5% of other CNS-associated macrophages were positive by flow cytometry. ^∗^3: choroid plexus macrophages express Siglec-H in steady state. ^∗^4: meningeal macrophages shows no or very faint Olfml3-positive. ^∗^5: especially positive on perivascular and meningeal macrophages. ^∗^6: infiltrating macrophages become positive. MG, microglia; MP, macrophages; ND, not detected.*

## Parenchymal Microglia, VAM, and PVMs

In a healthy brain, microglia reside in the parenchyma, while central nervous system (CNS) macrophages are non-parenchymal and located in boundary regions: perivascular spaces, meninges, and the choroid plexus. Microglia and CNS macrophages have different developmental origins and are suggested to exert distinct functions. In this review, we will discuss three types of CNS immune cells located around cerebral small vessels: (i) parenchymal microglia, not juxtaposed to vessels, (ii) VAM: parenchymal microglia juxtaposed to cerebral vessels, and (iii) PVM located in perivascular spaces.

### Parenchymal Microglia and VAM

Parenchymal microglia are the brain-resident immune cells and they play crucial roles in the development, maintenance of homeostasis, and diseases in the CNS. Microglia are crucial in brain development and regulate many mechanisms including synaptic pruning and maturation and angiogenesis (Arnold and Betsholtz, 2013). Under physiological conditions in adult life, microglia are constantly monitoring their surroundings thanks to their fine ramified motile processes (Nimmerjahn et al., 2005). Once microglia encounter harmful substances, such as infiltrating components from blood, burden abnormal proteins, or cell debris, they become activated to phagocyte these harmful substances or to protect the damaged cells (Hu et al., 2015). Furthermore, microglia can promote angiogenesis in both physiological (Foulquier et al., 2019) and pathological conditions such as ischemic stroke, AD, multiple sclerosis (MS), and Parkinson’s disease (PD) (Zhao et al., 2018), highlighting the important cross-talk of microglia with the cerebral vasculature (VAM). There is, however, a lack of molecular studies to differentiate VAM from the general population of parenchymal microglia, and we suggest that an advanced molecular characterization should be undertaken to reveal their true nature and assess their potential protective and/or deleterious functions in the context of cerebrovascular diseases.

### Perivascular Macrophages

**FIGURE 1:**
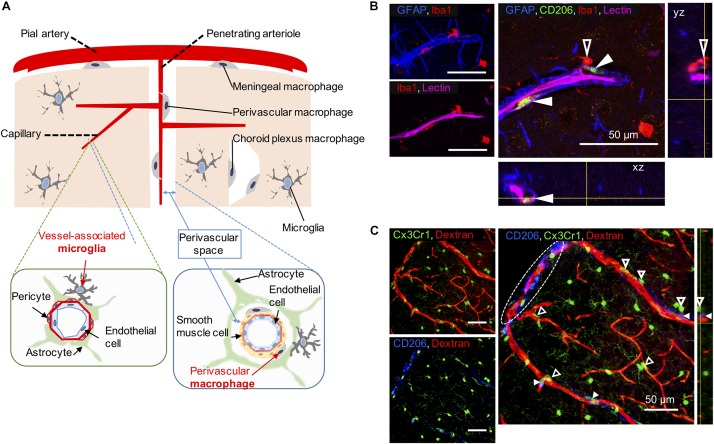
Vessel-associated microglia and perivascular macrophages (PVMs) in their workplace. **(A)** Scheme illustrating the differential location of CNS-macrophages including perivascular macrophages (PVMs), parenchymal microglia, and vessel-associated microglia (VAM) around the cerebral small vessels. Representative cortical areas in rat **(B)** and mice **(C)** imaged by confocal microscopy after immunostaining to reveal the presence and location of PVM (filled arrow heads) and VAM (empty arrow heads) using a set of different markers. **(B)** Two CD206-positive PVMs (green) are located between astrocyte end-feet (GFAP, blue) and endothelial cells stained by the injection of lectin. The PVM shows a flattened shape and a low-intensity Iba1 staining compared to VAM that are located beyond the glia limitans. The location of the *x*, *z* view (bottom) and the *y*, *z* view (right) corresponds to yellow lines. **(C)** Elongated CD206-positive PVMs (blue) are located along a large penetrating arteriole and pia artery (in white dotted circle) stained by the injection of 70-kDa dextran-Texas Red in a transgenic Cx3Cr1^gfp/wt^ mouse. VAM show a high Cx3Cr1 expression (green) compared to PVMs. The location of the *y*, *z* view (right) corresponds to yellow lines.

Apart from microglia, CNS macrophages are also involved in the maintenance of brain homeostasis, but their role is limited to its borders. CNS macrophages reside in the non-parenchymal perivascular space, subdural meningeal spaces, and choroid plexus spaces – namely, PVMs, meningeal macrophages (MMs), and choroid plexus macrophages (CPMs), respectively (Iadecola, 2017; Figure 1A). PVMs and MMs persist throughout the life of the organism due to their longevity and their capacity of self-proliferation, rather than the infiltration of peripheral myeloid cells. In contrast, CPMs mainly depend on blood-derived immigrating Ly6C^hi^ monocytes after birth (Goldmann et al., 2016). While microglia and macrophages share many functions and markers, previous studies have revealed differential marker expressions useful for their distinction (next paragraph, Table 1).

## Distinguishing Microglia From PVMs

Studies specifically investigating the differential functions of microglia (including parenchymal microglia and VAM) and PVMs are lacking due to the absence of steadfast experimental systems (Sevenich, 2018; Zhao et al., 2018). However, the use of single-cell RNA-seq analysis or mass cytometry have brought additional evidences confirming their differential roles. Gene expression analyses and histological studies have reported cell-specific markers: TMEM119 (Transmembrane protein 119), P2RY12 (P2Y purinoceptor 12), SALL1 (Sal-like protein 1), Siglec-H (Sialic acid-binding immunoglobulin-type lectins), and Olfm3 (Olfactomedin 3) as microglia-specific markers, and CD163 and CD206 as CNS-macrophage-specific markers (Table 1). Among the microglia-specific markers, none shows a high expression level stable throughout the entire microglia’s lifespan, suggesting that the dynamics of each marker should be considered.

During development, microglia (including VAM) and PVM originate from yolk-sac progenitors (Alliot et al., 1999; Ginhoux et al., 2010; Salter and Stevens, 2017). Recent work using a combination of fate mapping with single-cell RNA-seq and parabiosis experiments has shown that PVMs and MMs arise from yolk-sac hematopoietic precursors too, while CPMs have either an embryonic or adult hematopoietic origin (Goldmann et al., 2016). This new insight into the common origin of microglia, VAM, and PVM raises a new question on the exact time point when microglia diverge from CNS macrophages and which triggers this differentiation. While the emergence of parenchymal microglia was evidenced between embryonic day 9.5 and 12.5 by using *Cx3cr1*^GFP/WT^ mice (Goldmann et al., 2016), PVMs emerge at embryonic day 14.5 at the time of BBB closure (Wong et al., 2017; Li and Barres, 2018).

In adulthood, most functional markers are shared between microglia, monocytes, and macrophages, although their expression level may differ (Baufeld et al., 2018; Butovsky and Weiner, 2018). Ionized calcium-binding adapter molecule 1 (Iba-1) is a representative marker of both microglia and CNS macrophages. While Iba-1 intensity can be used to discriminate PVMs from VAM by immunofluorescence, low vs. high intensity, respectively (Faraco et al., 2016; Koizumi et al., 2019), its combination with additional markers is valuable (Figure 1). TMEM119 allows the specific identification of microglia from other immune cells (Satoh et al., 2016; Furube et al., 2018), however, its expression seems limited to mouse and human cells so far (Bennett et al., 2016). Siglec-H and Olfml3 are also highly expressed in microglia, whereas CPMs and MMs showed a very faint expression (Konishi et al., 2017; Neidert et al., 2018). CD163 seems a rather selective marker for PVMs (Kim et al., 2006). In addition, microglia have also been distinguished from CNS macrophages by their low CD45 and low CD206 expression levels, although this constitutes a less accurate identification method (Baufeld et al., 2018). Therefore, although more selective markers exist, microglia and PVMs have been mostly distinguished by using the following combination of markers: CD45^lo^CD11b^+^CD206^–^ for microglia and CD45^hi^CD11b^+^CD206^+^ for PVMs (Goldmann et al., 2016).

With aging or disease progression, both microglia and PVMs participate in inflammatory responses and their phenotypes are often assessed by the expression of specific cytokines or surface receptors. An increased expression of CD68, or a decreased expression of P2RY12/*P2ry12*, are for example associated with the acquisition of a pro-inflammatory phenotype (Rabinowitz and Gordon, 1989; Mildner et al., 2017; Jordão et al., 2019). As with other tissue-resident macrophages, microglia can be polarized and traditionally categorized into M1 (pro-inflammatory) and M2 (anti-inflammatory, resolving) phenotypes. However, it is now admitted that no clear boundaries can be drawn to characterize microglia/macrophage function and that a more refined phenotypic characterization should be used in new studies (Franco and Fernandez-Suarez, 2015; Ransohoff, 2016). Furthermore, one has to take into account that the expression of surface markers useful for the identification and distinction between microglia and PVMs can also vary due to their activation level. Indeed, while CD163 is normally specifically expressed by PVMs as described above, CD163-positive microglia have been observed in AD patients (Pey et al., 2014).

A list of markers to ease the distinction of microglia, including VAM, and CNS macrophages, including PVMs, is summarized in Table 1. In addition, PVMs and VAM are displayed in representative confocal pictures from cortical areas from a rat (Figure 1B) and from a mouse (Figure 1C). Their identification is based on their location (vessel-associated and inside/outside the glia limitans), morphology (ramified vs. flattened shape), and the expression levels of different surface markers (Iba1; Cx3Cr1; CD206, CD163). Furthermore their differential position in respect to the glial limitans has also been confirmed by electron microscopy (Onoda et al., 2014; Joost et al., 2019). As indicated in a recent review, PVMs are only present in association with arterioles and venules, but not with capillaries, as PVMs are located in the perivascular space between the abluminal surface of the endothelial vessel basement membrane and the parenchymal basement membrane on the glia-limitans side (Iadecola, 2017). These two basement membranes are however combined in capillaries, leaving no space for PVMs while VAM are still present. This implies that the contribution of perivascular immune cells to cerebrovascular dysfunction may differ with the vessel size and that PVMs and VAM should be studied separately.

## Perivascular Immune Cells and Cerebrovascular Dysfunction

While the contribution of microglia has been studied in various neurodegenerative disorders, their involvement in cerebrovascular diseases has been less studied. In particular, we aimed at summarizing the studies on perivascular immune cells (VAM, PVM) in the context of cerebrovascular dysfunction.

### Literature Search Method

**FIGURE 2:**
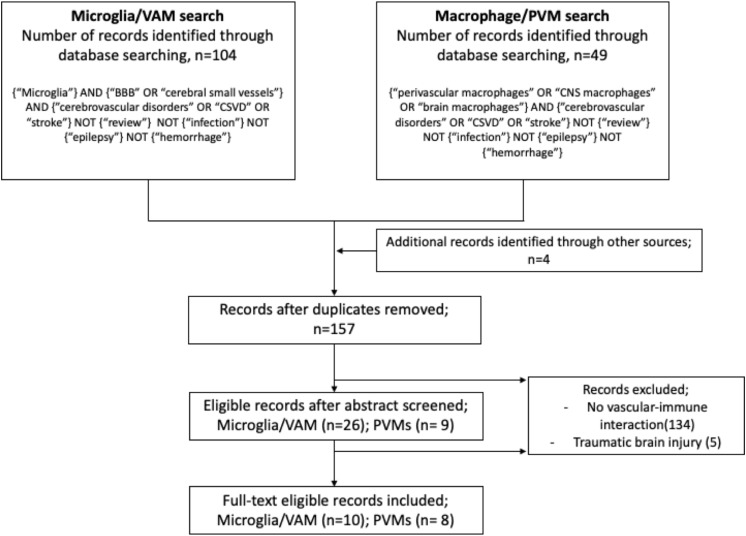
Flow diagram illustrating the systematic search protocol and the identification of the corresponding publication records.

Studies on VAM or PVMs and their involvement in cerebrovascular dysfunction were identified from electronic searches exclusively done by using the PubMed database. We used the following MeSH and free search terms to identify peer-reviewed original articles in English: for VAM: microglia AND (blood–brain barrier OR cerebral small vessel) AND (cerebrovascular disorders OR cSVD OR stroke); and for PVMs: (perivascular macrophages OR CNS macrophages OR brain macrophages) AND (cerebrovascular disorders OR cSVD OR stroke). In both searches, we excluded studies matching the following terms: review, infection, epilepsy, and hemorrhage. Furthermore, studies were excluded if they referred to microglia or macrophages in the context of brain tumor/metastasis, non-CNS diseases, infectious diseases, and drug or alcohol abuse. Screening and extraction of articles were done by TK under the guidance of SF. For each study, the following variables were recorded: (a) year of publication, (b) type of disease, (c) animal model, (d) microglia or macrophages markers, and (e) results. The searches were performed on October 30, 2019, and the results are described in a flow diagram (Figure 2).

### PVMs in Cerebrovascular Dysfunction

Perivascular macrophages have been shown to contribute to an increased vascular permeability and an increased granulocyte recruitment in the acute phase of stroke using the transient middle cerebral artery occlusion (tMCAO) model. Their depletion by the administration of clodronate-containing liposomes (CCLs) was indeed able to attenuate the vascular permeability, as evidenced by the reduced Evans blue extravasation 24 h post-ischemia, and to reduce the granulocyte infiltration in the ischemic cortex (Pedragosa et al., 2018). In an acute hypertension model induced by Angiotensin II infusion (2 weeks), the increased BBB permeability was shown to result from the generation of reactive oxygen species (ROS) mediated by the Angiotensin II type 1 receptor (AT_1_R) and the subsequent activation of Nox2 in PVMs. Indeed, the depletion of PVMs or the deletion of AT_1_R or Nox2 from PVMs, using bone marrow chimeras, were able to prevent BBB dysfunction, restore the neurovascular coupling and prevent cognitive dysfunction (Faraco et al., 2016). In SHR-SP, a chronic hypertensive model, PVM depletion with CCLs improved the endothelium-dependent dilation of the MCA, and prevented MCA structural remodeling induced by hypertension (Pires et al., 2014). Post-mortem investigation of brains from CADASIL patients (cerebral autosomal dominant arteriopathy with subcortical infarcts and leukoencephalopathy), a hereditary cSVD form, has revealed the presence of PVM-like cells with a phagocytic morphology around granular osmiophilic material depositions in arteries and arterioles, suggesting a role for PVMs to clear abnormal depositions in the perivascular space to prevent vascular remodeling (Yamamoto et al., 2013).

The contribution of PVM to cerebrovascular dysfunction was also evidenced in few studies on AD. PVM depletion using CCLs increased the number of amyloid depositions in cortical vessels of an AD mouse model an AD mouse model (Hawkes and McLaurin, 2009). This highlights again the crucial phagocytic role of PVMs to preserve the cerebrovasculature. This study reported for the first time that modulating PVM density can influence the clearance of amyloid from the cerebral vasculature. Unfortunately, it is highly possible that an increased clearance capacity can be accompanied by an increased ROS production. Park et al. (2017) have shown that the selective depletion of PVMs using intracerebroventricular injection of CCLs reduced ROS production and cerebrovascular dysfunction induced in different AD models. By using bone marrow transplantation, the involvement of CD36 and Nox2 from PVMs was demonstrated to contribute to the neurovascular dysfunction associated with the amyloid deposition (Park et al., 2017). These results indicate a crucial role for PVMs in clearing Aβ peptides from perivascular spaces and for preventing Aβ accumulation in cerebral vessels but it could be at the expense of a generation of ROS deleterious for the integrity of cerebral vessels in the long term.

In summary, while PVMs seem to exert protective effects at first to halt the progression of pathological events such as removal of harmful proteins, we suggest that their repeated activation and exposure to danger signals may lead over time to different brain disease-specific deleterious effects. The biology and pathobiology of PVMs in other brain diseases and in other tissues have been described in other recent reviews (Faraco et al., 2017; Lapenna et al., 2018).

### Microglia and VAM in Cerebrovascular Dysfunction

While the activation of parenchymal microglia in the presence of BBB leakages in hypertensive cSVD models is well known (Kaiser et al., 2014; Foulquier et al., 2018) evidences for the contribution of VAM to the initiation of cerebrovascular dysfunction remain limited.

In the genetic hypertensive rat model (Cyp1a1-Ren2), the microglia density increased in 6-month-old rats with a higher number of VAM (presumably VAM based on their shape, Iba1 immunoreactivity, and location), prior to any cerebrovascular lesions. This study further indicated that a modest but chronic blood pressure elevation can induce the regulation of growth factors and inflammatory genes prior to vascular remodeling, suggesting a role for VAM in the progression of cerebrovascular dysfunction (Pannozzo et al., 2013). In DOCA-salt rat, a sub-chronic hypertension model, while PVM phenotype did not change, VAM exhibited dynamic phenotypic changes; proliferative parenchymal and VAM proliferated before switching to a pro-inflammatory state and before BBB impairment and the occurrence of cerebrovascular lesions (Koizumi et al., 2019). In a post-mortem study on CAA, Carrano et al. revealed that major changes in tight-junction protein expressions (claudin-5, Occludin, ZO-1) were observed in CAA-affected capillaries engulfed by NADPH oxidase-2 (NOX-2)-positive activated microglia, and this was observed in association with BBB disruption (Carrano et al., 2012). In addition, Aβ induced ROS formation by binding RAGE (receptor of advanced glycogen product), an Aβ transporter. *In vitro*, blocking RAGE or inhibiting NOX-2 reduced the toxic effect of Aβ on endothelial cells (Carrano et al., 2011, 2012), supporting the evidence that the increased expression of NOX-2 in VAM could also affect the cerebral small vessels, similarly to the findings on PVMs.

Following an artificial BBB leakage induced by focal laser-injury, the immediate accumulation of processes from VAM toward the laser-injured capillary was capable of closing the BBB. This highly migratory behavior of VAM relied on the function of P2RY12 receptors as their inhibition using clopidogrel or their genetic ablation suppressed VAM motility and thereby led to a prolonged delay before BBB closure (Lou et al., 2016). This highlights the importance of VAM for BBB repair. In fact, the ultrastructural analysis of the laser injury by electron microscopy revealed that the aggregation of densely packed processes completely sheathed the site of injury. Immuno-labeling revealed that VAM processes, extending toward the laser-injured site, exhibited high P2RY12 expression (Lou et al., 2016). In this article, the authors used the term “juxtavascular microglia” to describe the vessel-associated Cx3Cr1-positive microglia at the capillary level with extended and fine processes, i.e., VAM. However, they wrongly defined juxtavascular microglia being largely localized within the perivascular space, which would correspond to the definition of PVMs. Another evidence has pointed toward a BBB repair role for VAM.

In tMCAO mice, Iba-1-positive cells with ramified processes start to cluster around vessels in the penumbra area within 1 h after the initiation of the ischemic insult. The attracted cells enwrap blood vessels in the penumbra 24 h post tMCAO and harbor an amoeboid shape and a high CD68 expression (Jolivel et al., 2015). The hypoperfusion induced by tMCAO in the area surrounding the ischemic core may continuously produce pro-inflammatory components, such as DAMPs, ROS, and inflammatory cytokines that may themselves, or after the leakage of plasma immunoglobulins or proteins, induce the infiltration of circulating monocytes or neutrophils into the brain parenchyma (Benjelloun et al., 1999; Krueger et al., 2019). The rapid mobilization of microglia after the ischemic insult suggests that VAM could be the first to initiate the BBB repair in the early phase of the injury before the migration of other parenchymal or systemic macrophages in the following hours/days.

In summary, parenchymal and VAM are quickly mobilized and accumulate around cerebral vessels following BBB dysfunction. In addition, they appear to be already present and activated in some conditions in the absence of BBB dysfunction in the early phase of cerebrovascular diseases and could therefore be targeted to prevent disease’s progression. This will however only be achieved thanks to a refined molecular characterization of their activation dynamics.

## Conclusion

The immune cells surrounding the cerebral vessels share many similarities, but their different locations suggest different functions. While recent studies have started to differentiate these two cell populations, their respective role and dynamics in the pathogenesis of cerebrovascular diseases are still unclear. This review aimed at summarizing the current direct and indirect evidences linking VAM and PVMs to cerebrovascular dysfunction. Our current knowledge on their role in BBB damage and repair is limited and should further integrate their dynamic nature. The emergence of transcriptomic and single-cell RNA sequencing techniques will lead to a more complete characterization and understanding of PVMs and VAM. Altogether, clarifying the roles of VAM and PVMs in physiological and pathological conditions may offer new perspectives for the diagnosis, prevention, and treatment of CNS diseases in which the vascular environment plays a crucial role.

## Author Contributions

TK, HS, and SF conceived the original idea. TK and SF wrote the manuscript with support from DK, TM, and HS.

## Conflict of Interest

The authors declare that the research was conducted in the absence of any commercial or financial relationships that could be construed as a potential conflict of interest.
